# Neurologic injuries after primary total ankle arthroplasty: prevalence and effect on outcomes

**DOI:** 10.1186/s13047-015-0112-7

**Published:** 2015-10-02

**Authors:** Andri Primadi, He-Xing Xu, Taek-Rim Yoon, Je-Hwang Ryu, Keun-Bae Lee

**Affiliations:** Department of Orthopedic Surgery, Chonnam National University Medical School and Hospital, 42 Jebongro, Donggu Gwangju, 501-757 Republic of Korea; Department of Orthopaedics and Traumatology, Universitas Padjadjaran Medical School/Hasan Sadikin Hospital, Bandung, Indonesia; Department of Pharmacology and Dental Therapeutics, School of Dentistry, Chonnam National University, Gwangju, Republic of Korea

**Keywords:** Neurologic injury, Total ankle arthroplasty, Prevalence, Outcomes

## Abstract

**Background:**

Neurologic injuries are complications that can arise after total joint arthroplasty. However, no comprehensive study has been conducted on peripheral nerve injuries after total ankle arthroplasty. The purpose of the present study was to identify the prevalence of neurologic injury following primary total ankle arthroplasty, the predisposing factors, and evaluate the effect on clinical outcomes.

**Methods:**

We retrospectively analyzed 150 consecutive primary total ankle arthroplasty using the mobile-bearing prosthesis between January 2005 and December 2011, in 150 patients with symptomatic ankle end-stage arthritis. All the patients were divided into groups according to whether they had postoperative peripheral neuropathy (23 patients) or not (127 patients). We investigated the prevalence, predisposing factors, and effect on clinical outcomes of neurologic injuries. The mean age was 61.3 years, and the mean follow-up period was 41.8 months.

**Results:**

There were 23 nerve injuries (15.3 %), including nine in posterior tibial nerves, six superficial peroneal nerves, six deep peroneal nerves, one saphenous nerve, and one sural nerve. Neurologic injury was significantly associated with the development of posttraumatic osteoarthritis, but it was not significantly associated with other predisposing factors, such as age, gender, body mass index, and symptom duration. Of the 23 nerve injuries, 13 (56.5 %) presented a complete, spontaneous recovery, 9 (39.1 %) presented an incomplete recovery, and 1 (4.3 %) presented no recovery. The patients with neurologic injury had significantly lower American Orthopaedic Foot and Ankle Society scores and lower levels of patient satisfaction.

**Conclusions:**

The results of this study suggest that the prevalence of neurologic injury after total ankle arthroplasty is considerable, and that neurologic injury is associated with low levels of patient satisfaction and poor clinical outcomes at mean of 3 years, postoperatively. Care is needed to reduce the occurrence of neurologic injuries.

## Background

Peripheral neurologic injury is an important complication following ankle arthroplasty, but its incidence is thought to be uncommon [1–7]. For lower limb arthroplasty, the common mechanisms related to neurologic injuries include traction, compression, entrapment, direct laceration, and indirect trauma [8–12]. Many studies have investigated the incidence of peripheral nerve injury following arthroplasty [9–12], and the prevalence of such injuries has been reported to range between 0.17 and 1 % after total hip arthroplasty [11, 13–16] and between 0.3 and 1.28 % after total knee arthroplasty [12, 17, 18].

Studies of neurologic injuries after total hip arthroplasty have identified the contributing factors to be traction injuries during positioning, prolonged traction, and traumatic compression as well as for the patient to be a female gender and for a less experienced surgeon to have carried out the procedure [14–16]. In total knee arthroplasty, the association between preoperative flexion contracture or valgus deformity and peroneal nerve injury has been established, but the effects of the application of a pneumatic tourniquet have yet to be conclusively determined [11].

In recent years, total ankle arthroplasty has become a viable option for patients with osteoarthritis of the ankle and has achieved favorable clinical results as it provides reliable pain relief, preserves motion, and facilitates recovery. Some studies reported the incidence of peripheral neurologic injuries in patients who underwent total ankle arthroplasty to be between 1.8 and 21 % of all cases, and these were found to be related to several causes [1, 10, 19–23]. Lee at al. [20] argued that the majority of nerve injuries were due to either excessive stretching during retraction or to improper release and improper protection distally in the incisional wound. However, no comprehensive study has been conducted on peripheral nerve injuries after primary total ankle arthroplasty.

The purpose of the present study was to evaluate the prevalence of neurologic injury following primary total ankle arthroplasty, identify predisposing factors (preoperative diagnosis, laterality and body mass index) for neurologic injury sustained during surgery, and evaluate effect of neurologic injury on clinical outcomes of American Orthopaedic Foot and Ankle Society (AOFAS) ankle-hind foot score, and patient satisfaction.

## Methods

We retrospectively analyzed 158 consecutive primary total ankle arthroplasty procedures, performed by a single surgeon between January 2005 and December 2011, in 158 patients with symptomatic ankle end-stage arthritis using the HINTEGRA prosthesis (Integra, Plainsboro, NJ, USA). We did not operate on patients with combined pathologies, such as charcot arthropathy, infectious arthropathy, and severe musculoskeletal injuries, since all of these can affect outcomes assessment. We excluded eight patients with preexisting sensoryor motor deficits (e.g., five diabetic peripheral neuropathy, three posttraumatic peripheral neuropathy). The remaining 150 patients were included in this study cohort. All the patients were divided into groups according to whether they had postoperative neuropathy (23 patients) or not (127 patients). This study was approved by the institutional review board of our hospital, and informed consent was provided to all patients.

There were 13 men (56.5 %) and 10 women (43.5 %), and the mean age was 54 years (33 to 70) in patients with a neurologic injury. And there were 78 men (61.4 %) and 49 women (38.6 %), and the mean age was 53 years (24 to 71) in patients without a neurologic injury. The average body mass index (BMI) was 24.5 kg/m^2^ in patients with neurologic injury and 25.6 kg/m^2^ in patients without a neurologic injury. Eighty-two patients had posttraumatic osteoarthritis, 65 presented primary osteoarthritis, and three patients had rheumatoid arthritis. We also checked the patient’s laterality and the symptom duration. The mean duration of the follow-up was 39 months (24 to 54) in patients with neurologic injury and 38 months (24 to 52) in patients without a neurologic injury (Table 1).

**Table 1:** Demographics of total ankle arthroplasty patients with and without neurologic Injuries

	With neurologic injury (*N* = 23)	Without neurologic injury (*N* = 127)	*p*-value^*^	RR (95 % CI)
Sex, male/female, n	13/10	78/49	0.495	0.98
Age, y	62.7 ± 10.5	61.1 ± 12.5	0.48	0.89
BMI^*^, kg/m^2^	24.5 ± 2.4	25.6 ± 3.2	0.130	0.99
Preoperative diagnosis, n				
Primary osteoarthritis	6	59	0.004	1.62
Posttraumatic osteoarthritis	16	66		
Rheumatoid arthritis	1	2		
Side of operation, Rt/Lt, n	14/9	77/50	0.771	0.74
Duration of ankle pain, m	38.7 ± 4.6	36.5 ± 3.4	0.167	0.84
Follow-up duration, m	44.7 ± 21.4	41.3 ± 21.7	0.767	0.98

*BMI* body mass index, *RR* releative risk, 95 % *CI* 95 % confidence

Values are expressed as mean ± SD unless otherwise indicated

^*^Independent *t*-test or Chi-square test. The *p*-values are of inter-group comparisons. Significance was accepted for *p*-values less than 0.05

To identify predisposing factors to nerve injury sustained during surgery, patients with and without neurologic injury were compared with regard to sex, age, BMI, preoperative diagnosis, operation laterality, symptom duration, and the preoperative AOFAS score [24].

Neurologic injuries that occurred as a complication from surgery were obtained by examining patients during their hospital stay or follow-up period, as written in their medical records. All patients were followed at one, three, six, and 12 months postoperatively, and annually thereafter. The data of clinical assessment included injured nerves, injury types, and recovery status.

**Fig. 1:**
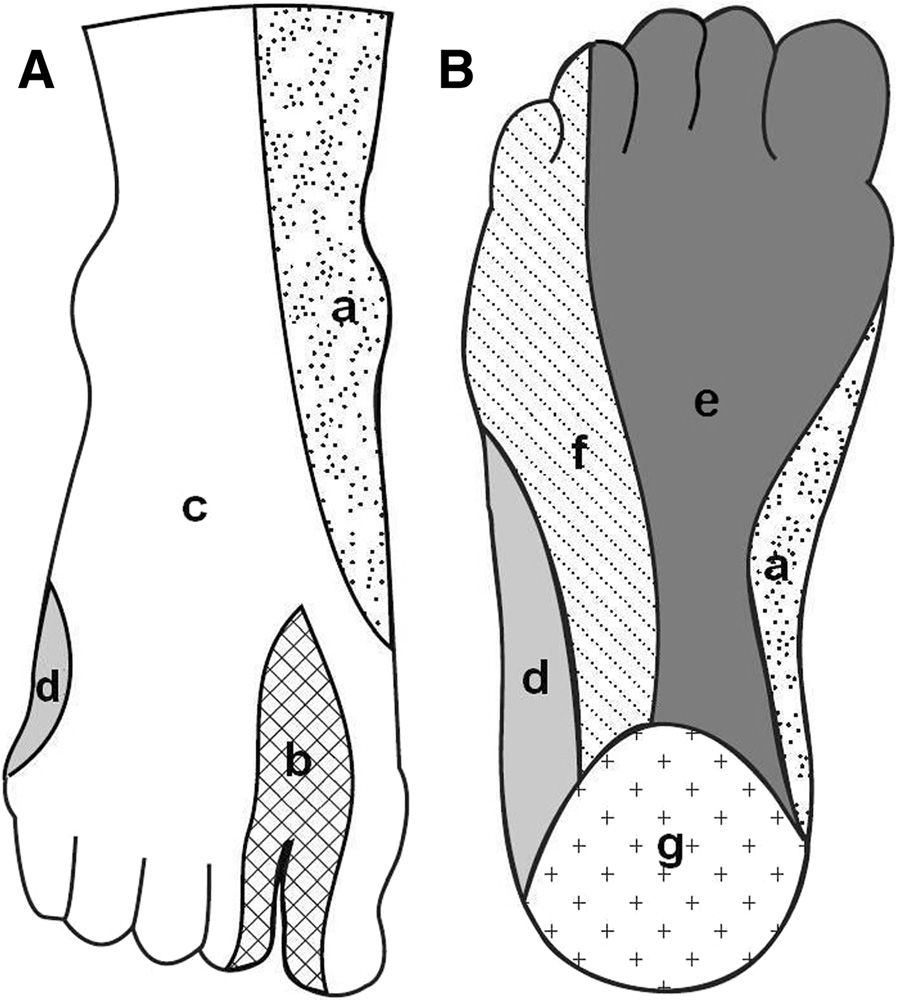
Cutaneous innervation of the foot. **a** Dorsal surface **b** Plantar surface. a. Saphenous nerve; b. Deep peroneal nerve; c. Superficial peroneal nerve; d. Sural nerve; e. Medial plantar nerve; f. Lateral plantar nerve; g. Medial calcaneal nerve

We used the clinical assessment to evaluate neurologic injuries according to cutaneous innervation of the foot (Fig. 1). For the patients in this study, electromyography studies were not routinely assigned. Injury types of nerve were classified as described by Asp and Randas complete or partial, sensory or motor, or a combination of these [9]. Any weakness in toe or ankle dorsiflexion was recorded as a motor deficit. Complete motor deficiency was defined to be when no contraction of innervated musculature occurred during examination, and partial motor deficit was defined to be when any muscle movement was retained. A profound loss of sensation was defined as a complete sensory deficit, and a less than complete loss was defined as a partial sensory deficit [9].

Patient recovery was graded as none, incomplete, or complete and was further evaluated in relationship to the baseline characteristics and perioperative conditions, such as the underlying diagnosis and additional procedures performed. Recovery of nerve injury is defined as aoccurance of skin sensation and muscle function. To avoid examiner bias, neurologic injury and clinical scoring were evaluated by two independent observers who were not involved in the surgical treatment of the patients.

For evaluating the effect of neurologic injury on clinical outcomes, the AOFAS score and patient satisfaction were used. The 100-point AOFAS scoring system combines subjective and objective data to evaluate clinical parameters; pain (40 points), function (45 points), and alignment (15 points). The AOFASscore is the most commonly reported outcome assessment after total ankle arthroplasty, but evidence of its validity and reliability is limited [25].

Furthermore, questionnaires were administered postoperatively to record patient satisfaction that was scored on a four-point Likert scale [26]. The response categories consisting of “very satisfied” (100 points), “somewhat satisfied” (75 points), “somewhat dissatisfied” (50 points), and “very dissatisfied” (25points). The scale score is the un-weighted mean of the scores from the individual items, ranging from 25 to 100 per item (with 100 being most satisfied).

### Statistical analysis

Descriptive statistics (arithmetic means, averages, and ranges) were calculated using standard formulas, to determine the significances of intergroup differences, univariable analysis was used to assess whether gender, age, preoperative diagnosis, BMI, operation laterality, duration of ankle pain and preoperative AOFAS score were predisposing factor for nerve injury. Multivariable linear regression analysis was used to assess the relationship between postoperative AOFAS score and above independent variables. The Paired *t*-test wasused to analyze AOFAS score intergroup and intragroup differences before and after surgery. And the Pearson’s Chi-square test or Fisher’s exact test was used to compare patient satisfaction between two groups. A *p*-value less than 0.05 was considered to indicate significance, and all aspects of the statistical analysis were reviewed by a statistician.

## Results

### Prevalence and typesof nerve injuries

The overall prevalence of nerve injuries was 15.3 % (23 patients) in 150 patients. Nine (39 %) of the 23 patients suffered from neurologic injuries that involved the posterior tibial nerve or one of its branches. There were six (26 %) cases where there was an isolated injury to superficial peroneal nerve, and in six (26 %) cases the injury was in the deep peroneal nerve. Sural nerve and saphenous nerve injuries were found in one (4 %) patient each. Almost all of them (19 out of 23, 82.6 %) presented partialsensory deficiency, two had both partial motor and sensory and two had complete sensory injuries (Table 2). Neurologic symptom was occurred at mean 2.1 months (range, 0.4 to 6.0 month) postoperatively.

**Table 2:** Summary of nerve injuries after primary total ankle arthroplasty

Injured Nerve	Number (%)	Recovery Status	Treatment	Patient Satisfaction
Posterior tibial	9 (39.1)	Complete : 3	Tarsal tunnel release: 3	Very satisfied: 3
		Incomplete : 5		Somewhat satisfied: 1
			Neurorrhapy: 2	
		None : 1	Observation: 4	Somewhat dissatisfied: 3
Superficial peroneal	6 (26.1)	Complete : 4	Observation: 6	Very dissatisfied: 2
		Incomplete : 2		Very satisfied: 4
				Somewhat satisfied: 2
Deep peroneal	6 (26.1)	Complete: 4	Observation: 6	Very satisfied: 4
		Incomplete: 2		Somewhat satisfied: 2
Sural	1 (4.3)	Complete: 1	Observation: 1	Very satisfied: 1
Saphenous	1 (4.3)	Complete: 1	Observation: 1	Very satisfied: 1

The cause for neurologic injury was estimated to be prolonged retraction or excessive nerve stretching, improper nerve release during surgery in 18 (78.2 %) cases. Tarsal tunnel syndrome was found in three patients who presented neurology at mean 4.3 month (1.0 to 6.0 month) postoperatively and posterior tibial nerve lacerations were observed in two patients who presented neurology at mean 3.2 month (0.9 to 6.0 month) postoperatively. Tarsal tunnel syndrome was confirmed by nerve conduction velocity and electromyography. Tarsal tunnel release and neurolysis were performed after failed conservative treatment including medication and physical therapy. Posterior tibial nerve lacerations were occurred by the saw blade during bone cuts at the posteromedial corner of the ankle without appropriate protection. The above five patients who had severe pain enough to interfere with the daily life or had obvious cause of pain underwent reoperation to treat neurologic injuries. Three patients with tarsal tunnel syndrome presented a partial sensory injury, and after decompression,1 recovered completely and two showed incomplete recovery. Two patients with posterior tibial nerve lacerations presented complete sensory injuries and underwent neurorrhaphy, but 1 showed incomplete recovery and 1 no improvement insymptoms.

### Predisposing factors

Neurologic injury had a significantly twice higher risk in patients with preoperative diagnosis of posttraumatic osteoarthritis (19.5 %) than patients with primary osteoarthritis (9.23 %) (*p* = 0.004) (Table 1, univariable analysis), but it was not significantly associated with other risk factors, such as age (*p* = 0.48), gender (*p* = 0.495), BMI (*p* = 0.13), laterality (*p* = 0.771), and duration of ankle pain (*p* = 0.167). Rheumatoid arthritis could not be considered during the analysis due to the lack of patients (0.02 %, 3 of 150).

To identify factors affecting postoperative AOFAS scores, patients were analyzed with multivariable regression analysis (Table 3). The preoperative diagnosis (*p* = 0.007) and nerve injury (*p* = 0.008) did present significant correlation to the postoperative AOFAS scores in both group, whereas gender (*p* = 0.508), age (*p* = 0.399), BMI (*p* = 0.917), laterality (*p* = 0.502), duration of ankle pain (*p* = 0.634) and follow-up duration (*p* = 0.604) did not. This study showed that the preoperative posttraumatic osteoarthritis and nerve injury may affect clinical outcomes on AOFAS score. Therefore, this study showed that preoperative diagnosis of posttraumatic osteoarthritis was considered to be the only significant predisposing factor for nerve injury among variables (Table 1, 3).

**Table 3:** Influence of individual factors on postoperative AOFAS in total ankle arthroplasty patients

	Estimate	(95 % CI)	*p*-value^*^
Sex, female	−0.976	(−1.936, 3.888)	0.508
Age, y	−0.049	(−0.165, 0.066)	0.399
BMI, kg/m^2^	−0.244	(−0.489, 0.441)	0.917
Preoperative diagnosis			
Posttraumatic osteoarthritis	1.389	(−1.768, 4.546)	0.007
Side of operation, Rt	−0.960	(−3.791, 1.870)	0.502
Duration of ankle pain, m	−0.128	(−4.165, 1.189)	0.634
Follow-up duration, m	0.017	(−0.048, 0.082)	0.604
Nerve injury	−5.419	(−9.421,−1.416)	0.008

*BMD* body mass index, 95 % *CI* 95 % confidence intervals

^*^Multivariable linear regression test. The *p*-values are of inter-group comparisons. Significance was accepted for *p*-values less than 0.05

### Recovery and clinical outcomes

At the final follow-up evaluation, 13 (56.5 %) patients presented a complete, spontaneous clinical recovery, nine (39.1 %) presented an incomplete recovery, and one (4.3 %) did not show any recovery. Most of the patients (18 of 23 patients, 78.2 %) recovered spontaneously. Five of 23 (21.8 %) patients underwent reoperation, and one (4.3 %) showed complete recovery, three (17.3 %) showed incomplete recovery, but 1 (4.3 %) had no recovery (Table 4).

**Table 4:** Literature review of neurologic injuries after total ankle arthroplasty

Study	Implant	Case (n)	Neurologic Injury, n (%)	Injured Nerve (n)	Recovery Rate	Risk Factors
Myerson et al. [21]	Agility	50	2 (4 %)	Deep peroneal (1)	0 (0 %)	Excessive stretching
				Superficial peroneal (1)		Improper release
						Improper protection
Knecht et al. [19]	Agility	69	15 (21 %)	Superficial peroneal (6)	N/A^a^	N/A
				Deep peroneal (3)		
				Common peroneal (6)		
Lee et al. [20]	Hintegra	50	3 (6 %)	Deep peroneal (3)	N/A^a^	Excessive stretching,
						Improper release
						Improper protection
Krause et al. [1]	Agility	114	2 (1.8 %)	Deep peroneal (2)	1 (50 %)	N/A-
	Mobility					
	STAR					
	Hintegra					
Current study	Hintegra	150	23 (15.3 %)	Posterior tibial (9)	22 (95.6 %)	Excessive stretching,
				Deep peroneal (6)		Improper release
				Superficial peroneal (6)		Improper protection
				Sural (1)		Laceration
				Saphenous (1)		

^a^N/A, data not available

The mean AOFAS score improved from 48.9 points (range, 22 to 58 points) preoperatively to 81.2 points (range, 66 to 90 points) at the final follow-up in the group with neurologic injuries (*p* = 0.002). In the neurologically intact group,the score improved from 47.9 points (range, 20 to 66 points) preoperatively to 88.4 points (range, 71 to 100 points) at the final follow-up (*p* = 0.001). There was a significant difference in the AOFAS score between the two groups at the final follow-up (*p* = 0.03).In particular, the two patients with a laceration of the posterior tibial nerve showed a poor AOFAS score (66 and 70 points in each) (paired t-test, *p* < 0.05).

With respect to the levels of patient satisfaction in the neurologically intact group, 115 patients (90.5 %) responded that they were either “Very satisfied” or “Somewhat satisfied,” three patients (7.8 %) responded to be “Somewhat dissatisfied,” and two patients (1.5 %) responded to be “Very dissatisfied”. In the neurologically injured group,18 patients (78.2 %) reported to be “Very satisfied” or “Somewhat satisfied”, three patients (13 %) reported to be “Somewhat dissatisfied”, and two patients (8.7 %) reported to be “Very dissatisfied”. There was a significant difference in the levels of patient satisfaction between the two groups at the final follow-up (fisher exact test, *p* = 0.017) . The patients with neurologic injuries had a lower reported satisfaction levels than those of the group without neurologic injury.

### Additional procedures

Eighteen additional procedures were carried out before or at the time of the total ankle arthroplasty to correct accompanying malalignment, joint contractures, or instabilities in three ankles (2.1 %) in the neurologically injured group and in 15 ankles (10.5 %) in neurologically intact group. Intraoperatively, percutaneous Achilles tendon lengthening was performed in one ankle in the neurologically injured group and in three ankles in the neurologically intact group. Deltoid release was performed in seven ankles only in the neurologically intact group. However, no significant difference with respect to these additional procedures was found between these two groups (Pearson’s Chi-square test, *p* = 0.934).

## Discussion

The most important finding of this study is that there were 23 nerve injuries (16 %), including nine posterior tibial nerves, six superficial peroneal nerves, and six deep peroneal nerves. Of the 23 patients, 13 (56.5 %) presented a complete recovery, nine (39.1 %) presented an incomplete recovery, and one (4.3 %) showed no recovery. The prevalence of a neurologic injury after primary total ankle arthroplasty was found to be considerable, and the neurologic injury is associated with low levels of patient satisfaction and poor clinical outcomes at a mean time of three years postoperatively.

In this study, overall rate (15.3 %)of peripheral neurologic injury was relatively higher than average rate (10.4 %)of the literature (Table 4). There was no report about any specific relation between the HINTEGRA prosthesis and prevalence of peripheral neurologic injuries. We supposed that was because we did accurate investigation interested in neurologic injury, including mild numbness which was often not reported in previous studies, at the time of every follow-up.

Generally, the precise etiology of a neurologic injury is rarely identified with absolute certainty. We found that posttraumatic osteoarthritis had a relationship with neurologic injuries sustained during surgery, and this might be logically explained by local scarring in the soft tissue that could tether the nerve, making it less able to tolerate even minor amounts of change [11]. A longer operation time is needed in such situations to ensure that a more cautious approach is taken, securing the operation field from more osteophytes, performing additional proceduresto correct deformities, and releasing scar tissue. Such conditions might be important causes of prolonged retraction of the nerve.

Our study did not find age, gender, BMI, symptom duration, or laterality to be significant risk factors for the development of nerve injury following total ankle arthroplasty. These findings were consistent with those presented by Rose et al. [27] who mentioned that patient age and gender were of no predictive value for the development of peripheral neuropathy after total knee arthroplasty. However some authors also postulated that female gender was a risk factors for neurologic complications, without presenting well established reasons [14].

Many authors have mentioned that, without appropriate release and protection, superficial and deep peroneal nerves are at risk of being injured by the saw during bone cuts, especially during cuts made at the dome of the talus [22]. We were very careful when the talus was cut to avoid injury of the superficial peroneal nerve, so there seems to have been no direct injury resulting from sawing the bone in our series. Direct pressure from a tight dressing, compression of the vascular supply to the nerve by the fascia, pressure from postoperative hematoma, and use of a tourniquet have been proposed to be possible cause as well [12]. In our institute, to avoid those kind of risk factors during and after operationthe adequate time for the tourniquet and meticulous hemostasis after release are kept, suction drainage is performed for 2 days after operation until there is only minimal drainage, and direct compression of the nerve under the swollen soft tissue is prevented by dressing the site with a cotton bandage.

In our study, 5 (21.7 %) patients out of 23 underwent reoperation to treat a nerve injury. The diagnosis consisted of three tarsal tunnel syndromes and two posterior tibial nerve lacerations. The other patients were each treated conservatively without surgical intervention. Ankle-foot orthosis and physical therapy involving range-of-motion exercises were used as required to further patient recovery. Generally, while minor stretch injuries may present spontaneous recovery, prolonged traction or traumatic compression of the nerve may show a less favorable prognosis, accounting for the higher percentage of permanent neurologic injuries in reoperation procedures that often require repeated manipulation of soft tissue [14].

We found three cases of tarsal tunnel syndrome following the surgery. Previous studies have described how traumatic injuries in the region of the tarsal tunnel can cause stenosis and scarring, leading to symptoms of the condition [4, 20, 21]. Entrapment resulting from space-occupying lesions has also been described. Bejjanki et al. [10] reported an unusual case that where the condition resulted from post-surgical entrapment and impingement from a displaced osteophyte rather than as a result of scarring. Extensive tibiotalar osteophytes are commonly seen and excised from patients undergoing ankle arthroplasty. It is difficult to ensure that all bony debris are removed from the surrounding soft tissue at the time of surgery.

Proper soft tissue release and familiarity with the surgical technique allowed us to avoid neurologic injuries [19, 20, 22]. In our series, the anterior approach was used in all cases. The anterior approach to the ankle is known to have a proximity to the deep peroneal nerve and to the branch of the superficial peroneal nerve, making them prone to injury. However, our results did not show any established relationship between this approach and an injury to the superficial/deep peroneal nerve. By handling the soft tissue carefully, an adequate incision can be made, and self-retaining retractors should be avoided because they carry the risk for uncontrolled retraction. Therefore, hand-retained retractors must be released intermittently during operation.

After a mean follow-up period of 41.8 months, 13 (56.5 %) of the patients showed a complete recovery of their symptoms, but nine (43.4 %) patients showed an incomplete clinical recovery, with 1 (4.3 %) showing no recovery. The potential for recovery often varies with the severity of the initial symptoms, and the patients’ comorbidities, peripheral vascular disease, and preexisting neuropathy should be assessed on a patient-by-patient basis [17].

This study has some limitations. First, the sample size and the number of patients who were discovered to have neurologic injuries were relatively small. This limits our ability to investigate the influence of baseline and perioperative characteristics to the outcome of interests. Second, we used a retrospective methodology. In the process of gathering analytical data of patients, even though author chose sample patients arbitrarily, a possible bias could occur. Moreover, we canonly presume for the cause of the neurologic injury to be excessive stretching, improper release and improper protection if there is no other identified cause, such as a laceration or tarsal tunnel syndrome. Third, operations for the present study were performed by a single surgeon in the same institute using the same prosthesis. This can be regarded as limitations on the interpretation of results for nerve injuries. Further investigation for multicenter trial is required. Finally, we did not routinely perform electro-diagnostic testing or nerve exploration. Instead,we used various clinical assessments of nerve injury, which have not yet been reported for their reliability and validity. Some authors have recommended routine electro-diagnostic testing for patients who are considered to be at high risk for sustaining a nerve injury during surgery. At our institution, we did not routinely monitor each patient. Currently, the indications for postoperative electromyography for the diagnosis and treatment of nerve injuries following total ankle arthroplasty have not been well established, and many patients recovered before electromyography was considered in a clinical situation.

Despite the limitations noted, this is the first comprehensive study evaluating the prevalence and types of neurologic injury following total ankle arthroplasty, and this is the largest series of nerve injuries following total ankle arthroplastyreported to date. As shown in this present study, patients with posttraumatic osteoarthritis are prone to nerve injuries after total ankle arthroplasty and showed low level of patient satisfaction and poor clinical outcomes. Therefore, surgery should be performed with care to avoid any nerve injuries.

## Conclusions

The conclusion of this study suggests that the prevalence of neurologic injury after primary total ankle arthroplasty is considerable, and that neurologic injury is associated with low patient satisfaction and poor clinical outcomes at a mean time of 3 years postoperatively. Therefore, care during surgery should be taken to reduce the occurrence of neurologic injuries.

## Abbreviations

AOFAS: American orthopedic foot and ankle society
